# Is CIS a Contraindication to Hyperthermic Intravesical Chemotherapy (HIVEC) after BCG-Failure?

**DOI:** 10.3390/cancers15051455

**Published:** 2023-02-24

**Authors:** Vassili Anastay, Michael Baboudjian, Alexandra Masson-Lecomte, Cédric Lebacle, Alexandre Chamouni, Jacques Irani, Xavier Tillou, Thibaut Waeckel, Arnaud Monges, Céline Duperron, Gwenaelle Gravis, Jochen Walz, Eric Lechevallier, Géraldine Pignot

**Affiliations:** 1 Department of Urology, La Conception Hospital, Aix-Marseille University, Assistance Publique des Hôpitaux de Marseille (APHM), 13007 Marseille, France; 2 Department of Urology, University de Paris, APHP, Saint Louis Hospital, 75010 Paris, France; 3 Department of Urology, Kremlin-Bicêtre Academic Hospital, 94270 Bicêtre, France; 4 Department of Urology, Centre Hospitalier Universitaire Caen, 14033 Caen, France; 5 Department of Urology, Polyclinique du Parc Rambot, Hôpital Privé de Provence, 13080 Aix-en-Provence, France; 6 Department of Urology, University Hospital François Mitterrand, 21000 Dijon, France; 7 Department of Medical Oncology, Institut Paoli Calmettes Cancer Center, 13009 Marseille, France; 8 Department of Surgical Oncology 2, Institut Paoli-Calmettes Cancer Center, 13009 Marseille, France; 9 Service de Chirurgie Oncologique 2, Institut Paoli-Calmettes, 232 Boulevard de Ste Marguerite, 13009 Marseille, France

**Keywords:** HIVEC, BCG failure, bladder cancer, NMIBC, CIS, recurrence, progression

## Abstract

**Simple Summary:**

Intravesical instillations of BCG remain the standard of care for high-risk non-muscle-invasive bladder cancer (NMIBC). In the case of BCG failure, radical cystectomy is recommended. When patients refuse to undergo BCG or are ineligible due to comorbidities, bladder-sparing techniques can be discussed, the majority of which are still being evaluated. Hyperthermic IntraVEsical Chemotherapy (HIVEC) in patients with carcinoma in situ (CIS) of the bladder remains controversial in terms of its oncological efficacy. In this multicentric retrospective study, including BCG-failed patients treated with HIVEC, we did not find increased recurrence or progression rates in patients with CIS. These data encourage further evaluation of HIVEC for the treatment of non-muscle-invasive bladder carcinoma regardless of the presence of CIS.

**Abstract:**

CIS of the bladder is associated with a high risk of progression. In the case of BCG failure, radical cystectomy should be performed. For patients who refuse or are ineligible, bladder-sparing alternatives are evaluated. This study aims to investigate the efficacy of Hyperthermic IntraVesical Chemotherapy (HIVEC) depending on the presence or absence of CIS. This retrospective, multicenter study was conducted between 2016 and 2021. Patients with non-muscle-invasive bladder cancer (NMIBC) with BCG failure received 6–8 adjuvant instillations of HIVEC. The co-primary endpoints were recurrence-free survival (RFS) and progression-free survival (PFS). A total of 116 consecutive patients met our inclusion criteria of whom 36 had concomitant CIS. The 2-year RFS rate was 19.9% and 43.7% in patients with and without CIS, respectively (*p* = 0.52). Fifteen patients (12.9%) experienced progression to muscle-invasive bladder cancer with no significant difference between patients with and without CIS (2-year PFS rate = 71.8% vs. 88.8%, *p* = 0.32). In multivariate analysis, CIS was not a significant prognostic factor in terms of recurrence or progression. In conclusion, CIS may not be considered a contraindication to HIVEC, as there is no significant association between CIS and the risk of progression or recurrence after treatment.

## 1. Introduction

Bladder cancer (BC) is the seventh most commonly diagnosed cancer in the male population worldwide [1]. Because risk factors, detection methods, and access to treatment vary from country to country, BC incidence and mortality rates are not homogeneous. At diagnosis, approximately 75% of BC are non-muscle invasive diseases [2]. Adjuvant therapy after bladder resection is required to prevent recurrence or progression. Treatment strategies for adjuvant therapy have not evolved for many years. Patients are classified into three risk groups (low-risk, intermediate-risk, and high-risk) and adjuvant treatment stratified by tumor risk is recommended. For intermediate risk, this is either intravesical chemotherapy with Mitomycin C (MMC) or immunotherapy with Bacillus Calmette and Guerin (BCG). For high risk, the recommended adjuvant treatment is BCG. Despite efforts to identify relevant biomarkers, clinicopathological factors remain the most important prognostic factors.

Carcinoma in situ (CIS) of the bladder is defined as a high-grade flat lesion confined to the mucosa. The presence of CIS is known to be a significant pathological prognostic factor in patients with non-muscle invasive bladder cancer (NMIBC) [3]. Indeed, CIS is a high-grade non-invasive malignancy with a high tendency to progress and spread cell carcinoma in the upper tract, prostatic urethra, and para-urethral ducts. The risk of muscle invasive progression in untreated patients with CIS is up to 50% [4]. It has been shown that CIS has pathologically similar characteristics to muscle-invasive bladder cancer (MIBC) and seems to be the most common precursor of MIBC [3]. However, CIS of the bladder exhibits a heterogeneous clinical behavior. Reliable factors predicting the disease course of NMIBC with CIS are unavailable. Molecular subtypes have the potential for prognostic stratification of muscle-invasive bladder cancer, while their value for CIS patients is unknown. Therefore, intravesical BCG instillations remain the standard of treatment for CIS, with the aim of preventing and delaying the risk of progression to muscle-invasive disease [5,6]. However, up to 50% of patients fail to maintain a response within 5 years after BCG, with early recurrences occurring in 10–15% of cases. The management of these BCG failures is a current challenge with the aim of enabling bladder preservation while avoiding progression to muscle infiltration. Although radical cystectomy remains the recommended treatment, some patients decline to undergo or are ineligible.

For patients for whom surgery is not an option due to comorbidities or refusal, therapeutic alternatives have been investigated. Hyperthermic intravesical chemotherapy either with the concomitant use of a bladder recirculation system (HIVEC-BRS) or using microwave bladder heating (Radiofrequency-Induced Thermochemotherapeutic Effect (RITE)) appears promising in high-risk NMIBC, especially in the setting of BCG shortage [7,8,9]. Introducing thermal energy into cancer cells can enhance the effect of chemotherapy. The effects of hyperthermia are multifactorial [10,11]. It inhibits neoangiogenesis by depriving the tumor of its vascularization. In addition, the lipoprotein cell membrane becomes porous, leading to increased intracellular concentrations of MMC. Moreover, direct effects on DNA include strand breaks, changes in transcription, replication and cell-division mechanisms [12]. Hyperthermia also stimulates an immune response through circulating heat shock proteins (HSP) that activate dendritic cells, T cells, and NK lymphocytes, triggering an antitumor response [13]. The combined effect on cancer cells would lead to natural cell death by apoptosis. Mytomycine C (MMC) is a stable molecule at high temperatures which increases its penetrance and cytotoxicity [14]. Hyperthermic MMC instillation using the bladder recirculation system (BRS) has been shown to result in a higher concentration of MMC in bladder cells compared to normothermic MMC instillation [15].

Hyperthermic intravesical chemotherapy is commonly offered for patients with BCG refractory high-risk NMIBC when surgery is not possible [5]. However, its effect on CIS is not well established and the previous data assessing the oncological results of this intravesical bladder-sparing strategy in the presence of CIS is controversial.

The aim of this study is to define whether concomitant CIS in NMIBC with BCG failure has an impact on the response to HIVEC.

## 2. Materials and Methods

### 2.1. Patients

This retrospective multicenter study was conducted between 2016 and 2021 in accordance with the principles of Good Clinical Practice and the Declaration of Helsinki. Patients were identified retrospectively from a prospectively maintained database that records all HIVEC therapies from seven referral centers in France. Patients treated for an intermediate or high-risk NMIBC without prior exposure to BCG were not analyzed in this study.

The cohort included patients aged ≥18 years with BCG unresponsive NMIBC, defined as NMIBC who have failed treatment with BCG according to the definition of BCG failures from the International Bladder Cancer Group [16]. The cohort included BCG refractory (i.e., recurrence occurring within six months after BCG immunotherapy), early refractory (i.e., recurrence occurring within six to twelve months after BCG immunotherapy) and intermediate relapsing patients (i.e., recurrence occurring more than one year after the start of BCG immunotherapy, but less than six months from the last BCG exposure for patients receiving maintenance therapy). All tumors were completely resected (TURB) before HIVEC treatment. Re-TURB was realized according to the histological findings of the first resection (pT1 on the initial resection had a systematic re-TURB). Patients with an incomplete BCG induction course, or with less than 6 months of follow-up after HIVEC, or with missing variables needed for outcome analysis were excluded from the final analysis. All indications for HIVEC were validated by a multidisciplinary team after a discussion with each patient regarding the potential benefits and side effects of all available treatment modalities for the management of BCG-failed NMIBC.

### 2.2. HIVEC

The HIVEC treatment protocol was similar across all participating centers. The first treatment occurred 4–6 weeks after transurethral resection of the bladder (TURB) or re-TURB if indicated. All instillations were performed with the Combat BRS V2.0 system, according to the manufacturer’s instructions (Combat medical, Wheathampstead, UK).

Before instillation, diuresis was reduced and urine was alkalinized. Instillation consisted of 40 mg of MMC diluted in 40 mL of distilled water which was heated extravesically to 41–43 °C and recirculated at 200 mL per minute at stable pressure for 60 min. The treatment consisted of an induction course of 6 to 8 weekly instillations without maintenance.

### 2.3. Follow-Up and Endpoints

Standard follow-up according to the current guidelines was planned. Early flexible cystoscopy was performed within six weeks after the last HIVEC instillation. Voided urine cytology was collected prior to the cystoscopy. Patients were then followed with flexible cystoscopy and urine cytology at 3-month intervals for the first 2 years and then every 6 months. In the case of a cystoscopy lesion, a TURB was scheduled.

The co-primary endpoints were recurrence-free survival (RFS) and progression-free survival (PFS). RFS was defined as the time from the date of the last TURB to the occurrence of bladder recurrence. PFS was defined as the time from the date of the last TURB to the occurrence of histologically confirmed muscle-invasive bladder cancer. Patients who did not experience a recurrence or progression were censored at the last follow-up.

### 2.4. Data Analysis

Descriptive statistics were carried out of the available variables according to the presence or the absence of CIS. Categorical variables were reported as frequencies and percentages (%) and compared by the Chi-square test, and continuous variables as medians and interquartile ranges (IQR) and compared by the Mann–Whitney test. Kaplan–Meier curves were used to illustrate RFS and PFS after treatment according to according to the presence or the absence of CIS. Rates of recurrence and progression were compared with the log-rank test. Multivariable Cox proportional hazards model was used to evaluate the association between CIS and the hazard of recurrence and progression. The models were adjusted for variables which had a *p* value < 0.10 in univariate analysis. All statistical analyses were performed using the R software Version 4.1.3 (R Foundation for Statistical Computing, Vienna, Austria). All tests were two sided with a significance level set at *p* < 0.05.

## 3. Results

### 3.1. Patient Population

A total of 116 consecutive patients met our inclusion criteria, of whom 36 (31.0%) had CIS (with or without associated papillary disease). The median patients’ age was 72 years (IQR 67–79). The distribution of EORTC risk groups was as follows: 27 intermediate-risk (23.3%), 65 high-risk (56%) and 24 very-high-risk (20.7%) NMIBC.

Forty-four (37.9%) TURB procedures were performed using photodynamic diagnosis with hexaminolevulinate. Table 1 summarizes the characteristics of the participants.

As shown in Table 1, patients with CIS had a shorter time to recurrence after BCG, with higher proportion of BCG-refractory patients (41.7% vs. 16.2%, *p* = 0.01). As expected, all CIS are high-grade diseases and patients with CIS were more likely to be classified as very-high-risk NMIBC (*p* = 0.01).

### 3.2. Recurrence-Free Survival

**Figure 1:**
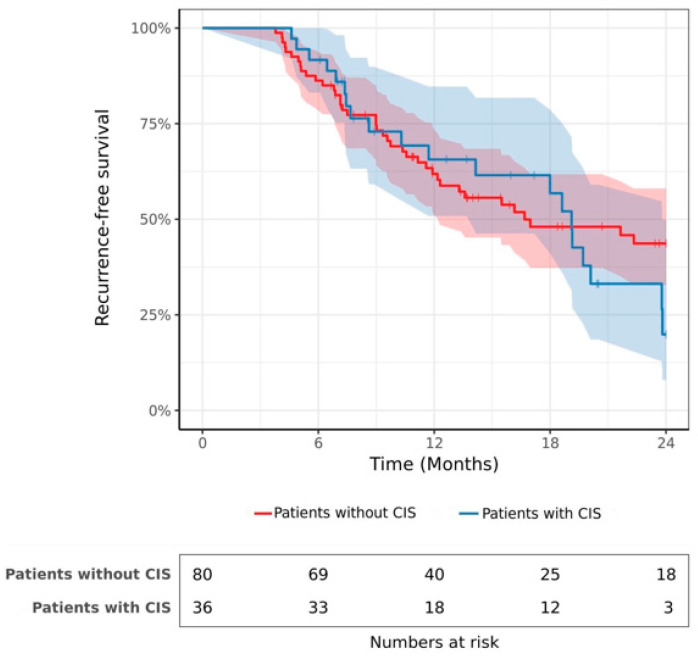
Recurrence-free survival after HIVEC in patients with and without CIS.

The median follow-up was 20.6 months. A total of 63 patients (54.3%) experienced bladder recurrence after a mean time of 16.6 months. The RFS rate was 62.9% at 1 year and 36.8% at 2 years. The estimated 2-year RFS rates were 19.9% (95% CI 0.07–0.50) and 43.7% (95% CI 0.32–0.58) in patients with and without CIS, respectively (Figure 1; log-rank *p* = 0.52). The results of the multivariable Cox hazards regression model are summarized in Table 2. No significant association was observed between CIS and the hazard of recurrence after HIVEC (HR 1.12, 95% CI 0.65 to 1.92; *p* = 0.7).

### 3.3. Progression-Free Survival

**Figure 2:**
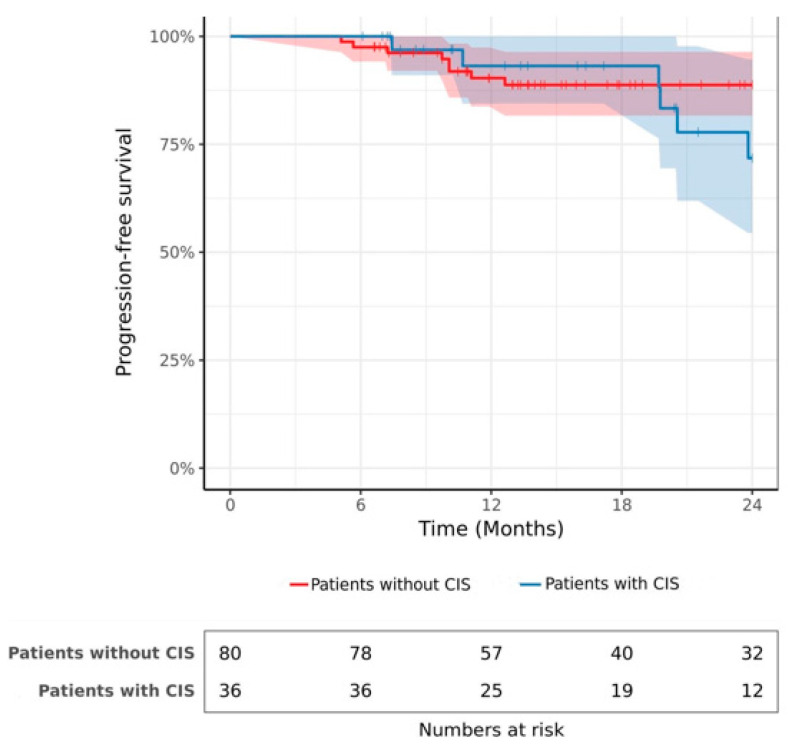
Progression-free survival after HIVEC in patients with and without CIS.

During the follow-up, a total of 15 patients (12.9%) experienced progression to muscle-invasive bladder cancer. The progression-free survival rate was 92.2% at 1 year and 87.9% at 2 years. The estimated 2-year PFS rates were 71.8% (95% CI 0.54–0.94) and 88.8% (95% CI 0.81–0.96) in patients with and without CIS, respectively (Figure 2; log-rank *p* = 0.32). The results of the multivariable Cox hazards regression model are summarized in Table 3. No significant association was observed between CIS and the hazard of progression after HIVEC (HR 2.65, 95% CI 0.74 to 9.44; *p* = 0.13). Only the T1 stage and tumor size > 3 cm were significant and independent prognostic factors associated with progression.

### 3.4. Safety

There were no severe adverse events (grade 3 or 4). Grade 1 or 2 adverse events consisted in hematuria (n = 10, 8.6%), bladder pain (n = 18, 15.5%), urgency (n = 17, 14.7%), myalgia (n = 1, 0.9%), bladder spasm or poor tolerance of the catheter during instillation (n = 13, 11.2%) and skin allergy (n = 6, 5.2%). No hospitalization was necessary due to adverse events. Eight patients (6.9%) had to stop the current treatment due to adverse effects. The ratio of delivered to planned instillations was 92%.

## 4. Discussion

MMC is a chemotherapy routinely used by urologists worldwide to treat NMIBC. Enhancement of the therapeutic effect by the use of hyperthermia has been demonstrated in the literature [17].

Regarding the results of HIVEC in patients with intermediate-risk NMIBC, two recent papers showed no benefit of hyperthermia. Angulo et al. did not find HIVEC to be superior to normothermic intravesical therapy for intermediate-risk NMIBC at 24 months [18]. These results are consistent with a phase 2, open-label, randomized controlled trial conducted by Tan et al., which found no significant difference in disease-free survival (DFS) between patients with intermediate-risk NMIBC treated with HIVEC and those treated with normothermic MMC [19]. Additionally, patients treated with HIVEC were more likely to experience treatment discontinuation and disease progression than those on the control arm. These two studies suggest that HIVEC should not be considered an adjuvant treatment option for intermediate-risk NMIBC. In contrast, in their randomized controlled trial, Colombo et al. provided encouraging results on the efficacy of heated MMC using RITE (Synergo^®^ system (Medical Enterprises, Amstelveen, The Netherlands) as an adjuvant approach for patients with intermediate and high-risk NMIBC, while excluding patients with CIS [20]. The results of this study showed that the heated MMC treatment was significantly superior to MMC alone. Indeed, the authors reported a 10-year disease-free survival rate of 53% for patients treated with RITE vs. 15% for those treated with MMC alone.

The question of whether this strategy could be discussed in the context of BCG-refractory patients is a current concern. First data assessed RITE (Radiofrequency Induced Thermochemotherapeutic Effect) by Synergo^®^ device with a randomized phase III study showing no significant benefit of microwave-heated chemotherapy (RITE) vs. standard treatment (re-challenge BCG in the majority of cases in this study) [21]. At 2 years, RFS in the RITE group was 35% vs. 41% in the BCG group (*p* = 0.49), with a non-significant improvement in the subgroup of patients with papillary tumor and no CIS (53% vs. 24%, *p* = 0.11). Conversely, patients with CIS (with or without associated papillary disease) had a significantly higher risk of recurrence in the RITE than in the control arm (2 years-RFS: 49% vs. 26%, *p* = 0.01). These data suggest that CIS could be a histological feature associated with treatment resistance. In a retrospective multicenter study assessing RITE in CIS patients, a 6-month CR rate of 46.0% in BCG-unresponsive patients was shown (vs. 83.0% in BCG-naïve patients) and a subsequent 2-year RFS rate of only 17.4% [22]. Since then, the majority of prospective randomized studies decided to exclude CIS from the selection criteria, in particular those evaluating HIVEC [23].

While HIVEC is commonly used to treat patients with high-risk NMIBC after BCG failure, its effect on CIS is not well established. The aim of this retrospective multicenter study was to assess the efficacy of HIVEC on CIS compared to papillary tumors without associated CIS. This work sheds light on the fact that HIVEC could be an alternative to radical cystectomy for NMIBC with concurrent CIS. Indeed, our results show no significant difference in progression or recurrence rates in patients with or without CIS, treated with HIVEC after BCG failure. The estimated 2-year PFS rate of 71.8% in the CIS population indicates that HIVEC may be safely discussed as an alternative option in these patients ineligible or who refuse cystectomy.

In their retrospective study, Pijpers et al. investigated the long-term effects of HIVEC with the same protocol as ours in 56 patients who did not respond to BCG [24]. After a median follow-up of 32.2 months, the 1- and 2-year high-risk-RFS was 53% and 35%, respectively, with no significant difference between CIS and non-CIS patients. Similarly, in a post hoc analysis of 55 BCG unresponsive patients, De Jong et al. did not find any difference in terms of RFS between CIS and non-CIS patients [25]. These results, quite similar to ours, give us confidence in treating CIS with HIVEC in BCG-failed NMIBC. These findings may help further studies to compare standard treatment with HIVEC in high-risk patients including CIS which has often been excluded from controlled trials.

Notably, 12.9% of patients had progressed to a muscle-invasive disease during follow-up. The risk of tumor progression is thus considered in patients who do not respond to therapy. Moreover, it has been shown that patients who progress from a high-risk NMIBC such as CIS to a muscle-invasive tumor have a significantly worse prognosis than patients with a primary muscle-invasive tumor [26].

In a retrospective study, Huang et al. analyzed the survival of patients who underwent radical cystectomy with pelvic lymph node dissection for CIS-only BC. The estimated 5-year overall survival and recurrence-free survival were 87 and 100%, respectively [27]. These results underline that the oncological results of radical surgery remain better than those of HIVEC, even if associated with poor functional outcomes [28]. Regarding the risk of progression, which is an important endpoint in this setting of bladder preservation, patients should, therefore, be well-informed of the risk of undertreatment when refusing cystectomy with a potential risk of putative progression to muscle-invasive disease, which could have a significant impact on survival, despite the initiation of HIVEC treatment.

Other intravesical drugs for BCG failure include other chemotherapies such as epirubicin or valrubicin, but also vicinium, and electromotive drug administration [29]. Electromotive drug delivery (EMDA^®^-MMC, Physion SRL, Mirandola, Italy) is one of the techniques to increase the depth of penetration. There are currently no direct comparisons between EMDA^®^-MMC and hyperthermic strategies such as RITE or HIVEC, either in terms of relative penetration into the bladder wall or in terms of oncological outcomes. Regarding the prognostic value of CIS, response rates to EMDA^®^-MMC were significantly worse in the presence of CIS, with a disease-free survival rate of only 50% at 9 months in this subgroup of patients [30].

More recently, encouraging clinical trial results for alternative intravesical therapies have been released in this setting of BCG-unresponsive population [29]. Nadofaragene firadenovec, an IFN-based gene therapy, showed promising results in patients with CIS, with a 53.4% complete response rate at 3 months, of which only 45.5% had a sustained response to 12 months [31]. Oportuzumab monatox, an Antibody–Drug Conjugate, and IL15 superagonist N-803 that enhance tumor targeting in combination with BCG, also showed encouraging first data [32,33]. In a recent meta-analysis, a lower response rate of these post-BCG bladder-sparing strategies was reported in patients with CIS [29].

Novel treatment modalities also include systemic strategies, in particular immunotherapy. In these patients with BCG-refractory CIS (with or without papillary disease), preliminary results from studies such as SWOG S1605 (NCT02844816) or Keynote-057 (NCT02625961) show poor oncological outcomes while significant adverse events [34,35]. In the Keynote-057 trial, Pembrolizumab achieved a complete response rate at 3 months of 38.8%, which led the FDA to approve its use in January 2020 [35]. However, half of the responder patients relapsed within the first year, resulting in a 1-year RFS rate of less than 20%. Moreover, toxicities are not negligible with these systemic immunotherapies compared to intravesical strategies. Results from ongoing trials will provide us with useful information about many of the existing regimens and probably new drugs will soon be available for this group of patients.

Strengths of our study include the use of data that is prospectively registered from seven referral centers in France. To our knowledge, this study is the largest series evaluating the efficacy of HIVEC in a well-defined cohort of patients with BCG failure, including CIS.

The main limitations of our study include the small number of CIS patients, the retrospective design, and the lack of photodynamic diagnosis (PDD) cystoscopy and systematic biopsies after HIVEC. Indeed, new optical techniques have been shown to improve the detection of CIS in the bladder. Because CIS is a flat tumor, PDD in addition to white light cystoscopy allows better detection of recurrences [36,37]. Earlier detection of recurrences could authorize better management of relapsing NMIBC. Finally, the population is quite heterogeneous, with some patients having received only the induction course of BCG while others have had a maintenance regimen. Pure CIS vs. CIS associated with a papillary tumor could not be analyzed separately, although they are probably two different entities of the disease.

A further investigation targeting CIS response to HIVEC, especially on a molecular level, should be conducted to better identify patients likely to respond to treatment and those at high risk of progression.

## 5. Conclusions

Our work shows that concurrent CIS in NMIBC with BCG failure should not be considered a contraindication to HIVEC treatment, as there is no significant association between CIS and the risk of progression or recurrence after treatment. HIVEC is a well-tolerated and safe bladder-sparing treatment and could be discussed as an alternative to cystectomy for BCG-failure patients ineligible or refusing it, even if CIS is present. Prospective and collaborative studies should be conducted to confirm our findings.

## Figures and Tables

**Table 1:** Baseline Characteristics.

	Patients without CIS (n = 80)	Patients with CIS (n = 36)	*p*
Age	72 (66–79)	74 (69–79)	0.7
Time to recurrence after BCG			**0.01**
<6 mo	13 (16.2)	15 (41.7)	
6–12 mo	19 (23.8)	7 (19.4)	
>12 mo	48 (60)	14 (38.9)	
Tumor size			>0.9
<3 cm	72 (90)	33 (91.7)	
≥3 cm	8 (10)	3 (8.3)	
Tumor number			0.08
Unique	36 (45)	10 (27.8)	
Multiple	44 (55)	26 (72.2)	
T1 stage	25 (31.1)	8 (22.2)	0.3
Tumor grade			**0.01**
Low	26 (32.5)	0	
High	54 (67.5)	36 (100)	
PDD use	30 (37.5)	14 (38.9)	0.9
EORTC-risk groups			**0.01**
Intermediate-High	69 (86.3)	23 (63.9)	
Very High	11 (13.7)	13 (36.1)	
Detrusor muscle presence	58 (72.5)	27 (75)	0.8
reTUR performed	26 (32.5)	10 (27.8)	0.6

Data are presented as median and interquartile ranges, or as numbers and percentages. Bold for significant value. BCG: Bacillus Calmette–Guerin, PDD: photodynamic diagnosis with hexaminolevulinate, EORTC: European Organisation for Research and Treatment of Cancer, re-TUR: second-look transurethral resection.

**Table 2:** Multivariable results of Cox proportional Hazards Regression to predict bladder recurrence after HIVEC.

Variables	Bladder Recurrence		
	HR	95% CI	*p*
Carcinoma in situ	1.12	0.65–1.92	0.7
Tumor size			
<3 cm	Ref.	Ref.	Ref.
≥3 cm	1.93	0.90–4.11	0.09
Detrusor muscle presence	1.83	0.98–3.40	0.06

Patient age, time to recurrence after BCG therapy, T stage, tumor grade, tumor number, photodynamic diagnosis use, EORTC risk groups and maintenance therapy were not included in the multivariate model as they had a *p*-value > 0.10 in univariate analysis.

**Table 3:** Multivariable results of Cox proportional Hazards Regression to predict bladder progression after HIVEC.

Variables	Bladder Progression		
	HR	95% CI	*p*
Carcinoma in situ	2.65	0.074–9.44	0.13
T stage			
Ta	Ref.	Ref.	Ref.
T1	16.5	3.19–85.2	**<0.001**
Tumor size			
<3 cm	Ref.	Ref.	Ref.
≥3 cm	7.57	1.70–33.6	**0.008**
EORTC risk group			
Intermediate-High	Ref.	Ref.	Ref.
Very High risk	0.27	0.04–1.71	0.2

Patient age, time to recurrence after BCG therapy, tumor grade, tumor number, photodynamic diagnosis use and maintenance therapy were not included in the multivariate model as they had a *p*-value > 0.10 in univariate analysis. Bold for significant value.

## Data Availability

Data supporting reported results can be obtained with courtesy of ethics committee of the Paoli-Calmettes Institute and in accordance with privacy or ethical restrictions.
